# Cortical control of a tablet computer by people with paralysis

**DOI:** 10.1371/journal.pone.0204566

**Published:** 2018-11-21

**Authors:** Paul Nuyujukian, Jose Albites Sanabria, Jad Saab, Chethan Pandarinath, Beata Jarosiewicz, Christine H. Blabe, Brian Franco, Stephen T. Mernoff, Emad N. Eskandar, John D. Simeral, Leigh R. Hochberg, Krishna V. Shenoy, Jaimie M. Henderson

**Affiliations:** 1 Department of Neurosurgery, Stanford University, Stanford, CA, United States of America; 2 Department of Electrical Engineering, Stanford University, Stanford, CA, United States of America; 3 Department of Bioengineering, Stanford University, Stanford, CA, United States of America; 4 Neurosciences Institute, Stanford University, Stanford, CA, United States of America; 5 Bio-X Institute, Stanford University, Stanford, CA, United States of America; 6 Neurosciences Program, Stanford University, Stanford, CA, United States of America; 7 School of Engineering, Brown University, Providence, RI, United States of America; 8 Carney Institute for Brain Science, Brown University, Providence, RI, United States of America; 9 Center for Neurorestoration and Neurotechnology, Rehabilitation Research and Development Service, VA Medical Center, Providence, RI, United States of America; 10 Department of Biomedical Engineering, Emory University and the Georgia Institute of Technology, Atlanta, GA, United States of America; 11 Department of Neurosurgery, Emory University, Atlanta, GA, United States of America; 12 Department of Neuroscience, Brown University, Providence, RI, United States of America; 13 Center for Neurotechnology and Neurorecovery, Department of Neurology, Massachusetts General Hospital, Boston, MA, United States of America; 14 Department of Neurology, Warren Alpert Medical School of Brown University, Providence, RI, United States of America; 15 Department of Neurosurgery, Harvard Medical School, Boston, MA, United States of America; 16 Department of Neurosurgery, Massachusetts General Hospital, Boston, MA, United States of America; 17 Department of Neurology, Harvard Medical School, Boston, MA, United States of America; 18 Department of Neurobiology, Stanford University, Stanford, CA, United States of America; 19 Howard Hughes Medical Institute at Stanford University, Chevy Chase, MD, United States of America; Shanghai Jiao Tong University, CHINA

## Abstract

General-purpose computers have become ubiquitous and important for everyday life, but they are difficult for people with paralysis to use. Specialized software and personalized input devices can improve access, but often provide only limited functionality. In this study, three research participants with tetraplegia who had multielectrode arrays implanted in motor cortex as part of the BrainGate2 clinical trial used an intracortical brain-computer interface (iBCI) to control an unmodified commercial tablet computer. Neural activity was decoded in real time as a point-and-click wireless Bluetooth mouse, allowing participants to use common and recreational applications (web browsing, email, chatting, playing music on a piano application, sending text messages, etc.). Two of the participants also used the iBCI to “chat” with each other in real time. This study demonstrates, for the first time, high-performance iBCI control of an unmodified, commercially available, general-purpose mobile computing device by people with tetraplegia.

## Introduction

Millions of people have some form of paralysis, which can limit the ability to perform activities of daily living [1]. In conditions such as amyotrophic lateral sclerosis (ALS), the disease may eventually impede both speech and other forms of effective communication [2]. The field of assistive technology aims to improve the functional capabilities of people with disabilities [3]. Augmentative and alternative communication (AAC) interventions are standard-of-care for people with complex communication impairments [3, 4]. An emerging input method for AAC or other technologies is a brain-computer interface (BCI), which translates brain activity into useful control signals for computing devices. Using BCIs based on electroencephalography (EEG, which records signals from the scalp) [5–9] or electrocorticography [10], previous work has shown control of spelling, web browsing, games, and painting [11–14]; but not general control of a computing device such as a commercial tablet. Similarly, intracortical BCIs have demonstrated compelling proofs-of-principle in both preclinical [15–24] and clinical [25–33] trials but, to date, there has been no demonstration of high-performance control of familiar applications on unmodified consumer computing devices. In this study, we tested the feasibility of using an iBCI enabled by advanced neural decoders [24, 31, 32, 34], building primarily on a recent prior report [33] to provide “point-and-click” control of a commercial tablet computer by three people with limited arm and hand movement.

## Materials and methods

### Permissions

Permission for these studies was granted by the US Food and Drug Administration (Investigational Device Exemption) and the Institutional Review Boards of Stanford University, Providence Veterans Affairs Medical Center, Brown University, and Massachusetts General Hospital. The participants in this study were enrolled in a pilot clinical trial of the BrainGate2 Neural Interface System (ClinicalTrials.gov Identifier: NCT00912041).

### Participants

Participants were enrolled according to the inclusion and exclusion criteria of the clinical trial, and informed consent was obtained for all study-related protocols and procedures. Separate consent to publish photos and video was also obtained.

Participant T6 is a right-handed woman, 51 years old at time of study enrollment, diagnosed with ALS and with resultant motor impairment. In December 2012, a 96-channel intracortical microelectrode array (1.0-mm electrode length, 4 × 4 mm, Blackrock Microsystems, Salt Lake City, UT) was placed in the hand area of dominant motor cortex as previously described [26, 34]. At the time of this study, T6 retained speech and dexterous movements of her wrists and some fingers (ALSFRS(R) = 14). Data reported in this study are from T6’s post-implant trial days 1013, 1018, and 1034.

Participant T9 was a right-handed man, 51 years old at time of study enrollment, also diagnosed with ALS. In February 2015, he had two microelectrode arrays (1.5-mm electrode length, same manufacturer) placed in the hand area of dominant motor cortex. At the time of this study, T9 retained speech and had minimal and nonfunctional movement of the fingers (ALSFRS(R) = 6). Data reported in this study are from T9’s post-implant trial days 218, 222, and 225.

Participant T5 is a right-handed man, 63 years old at the time of study enrollment, with tetraplegia due to a C4 ASIA C cervical spinal cord injury. In August 2016, he had two microelectrode arrays (1.5-mm electrode length, same manufacturer) placed in the hand and arm area of dominant motor cortex. At the time of this study, T5 retained speech and had minimal and nonfunctional movement of the fingers. Data reported in this study are from T5’s post-implant trial days 121, 124, and 140. A fourth session (post-implant trial day 126) was also attempted, but was unsuccessful because of a cable malfunction (which was subsequently remedied).

### Research setup

**Fig 1:**
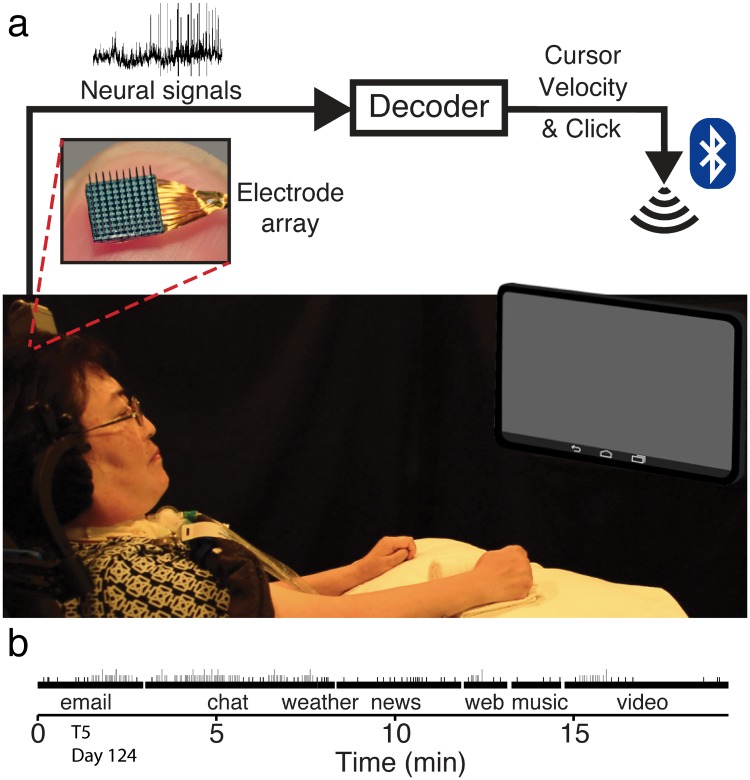
Research setup. **a** Schematic of research setup with T6. We recorded from 96-channel electrode arrays implanted in motor cortex. The neural signals extracted from the arrays were passed into a decoding algorithm which output a two dimensional cursor velocity and a click signal. The output of the decoder was presented as a wireless Bluetooth mouse interface and paired with a computer tablet. The participants used this interface to control the tablet and perform common tasks like email and web browsing. **b** Example task timeline with T5 from trial day 124. Shortest vertical black lines represent general user interface clicks, shorter gray lines represent single character text entry, and taller gray lines represent autocompletion of text.

The research setup was similar to prior reports [26, 31–33, 35] for the purposes of data recording, processing, and analysis. A NeuroPort recording system (Blackrock Microsystems, Salt Lake City, UT) recorded neural signals from the participant’s motor cortex. These signals were routed into a custom real-time computer running the xPC/Simulink Real-Time operating system (Mathworks, Natick, MA) for processing and decoding. The output of the decoding algorithm was passed to a Bluetooth interface configured to work as a conventional wireless computer mouse using the Bluetooth Human Interface Device (HID) Profile. This virtual Bluetooth mouse was paired with a commercial Android tablet device (Google Nexus 9, Android OS 5.1) with no modifications to the operating system. Each participant viewed the device at their preferred comfortable distance, typically 40-60 cm from the eyes. No accessibility software was installed on the tablet, and no built-in accessibility features were enabled. Participants performed real-time “point-and-click” control over a cursor that appeared on the tablet computer once paired through the Bluetooth interface. Fig 1a details the flow of information from the participant to the tablet device. Advanced cursor features such as click-and-hold, multitouch, and gestures were not implemented in this study.

Videos of the study were captured in two ways. An external DSLR camera was positioned to record the participant as they controlled the tablet. Simultaneously, a screen capture program (AZ Screen Recorder, Hecorat) running on the tablet recorded all activity on the tablet as a video.

### Neural decoders

In this study, intended cursor movements and clicks were decoded from neural activity using Kalman filters for cursor movement and state classifiers for click detection. 2D cursor velocities were estimated using a Recalibrated Feedback Intention Trained Kalman Filter (ReFIT-KF) for T6 and T5 [21, 31, 33] and a cumulative closed-loop decoder for participant T9 [35]. Briefly, the ReFIT-KF is a decoder built in a two-step fashion which attempts to correct the kinematics of first-pass iBCI control by assuming intention to move directly to the target, leading to improved performance. The cumulative closed-loop decoder is typically initialized using neural data recorded during an open-loop task. Additional data, recorded during closed-loop neural control, are then used to update decoder parameters, with the aim of refining the tuning model [35]. In order to reduce calibration time, it is also possible to seed the decoder with parameters from the previous research session, as was the case on T9’s trial days 222 and 225. Different decoders were used in this study because we aimed to highlight iBCI reliability and robustness. Being relatively decoder agnostic demonstrates that the performance achieved here is not intricately linked to the specifics of a single decoder, but that multiple decoding approaches can successfully drive a common communication device. Click intentions were classified using a hidden Markov model for T6 and T5 [24, 33] and a linear discriminant analysis classifier for T9 [34]. Participants each had their own imagery to enact a click. T6 attempted squeezing her left hand T5 attempted flexing his left arm. T9 attempted squeezing his right hand. The duration of the calibration blocks (excluding voluntary participant pauses between blocks) used to initialze the decoder, in minutes, for each day and each participant were: T6 (10, 12, 8), T9 (25, 4, 12), and T5 (20, 12, 16). Methods for further reducing this initial calibration period have been implemented more recently [36].

To initialize and calibrate the decoders, participants engaged in a center-out-back task described previously [31, 33, 35]. These decoders were built in a stepwise fashion, with the first stage of filter calibration performed as the cursor moved automatically to the targets while the participants imagined or attempted moving their hand as though they were controlling the cursor. This allowed the initialization of a decoder that was then improved upon in subsequent calibration blocks. The Kalman filters were also running bias correction algorithms throughout the task [32]. For T6 and T5, once core data collection began (see below), there were no decoder modifications or interruptions aside from voluntary inter-task breaks. Decoder bias re-estimation blocks were permitted as needed during the free-time period that followed core data collection when T6 and T5 were using the tablet to explore their interests. For T9, no decoder modifications or interruptions aside from voluntary inter-task breaks were performed once he started using the tablet.

**Fig 2:**
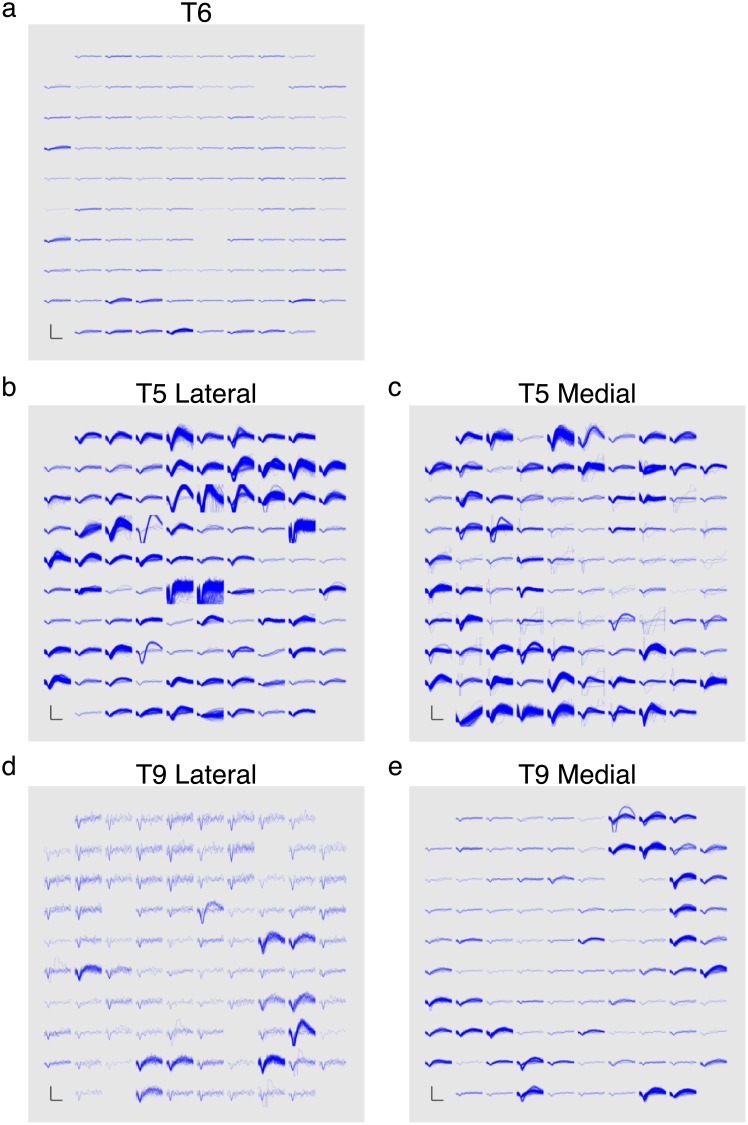
Thresholded spiking actvity of participants’ arrays. Each panel, corresponding to a specified 96-channel array, shows the threshold crossing waveforms recorded over 60 seconds on the specified trial day. **a** is T6’s array. **b** and **c** are T5’s lateral and medial arrays, respectively. **d** and **e** are T9’s lateral and medial arrays, respectively. Scale bars represent 150 uV (vertical) and 500 us (horizontal). Data are from the following trial days: 1013 (T6), 124 (T5), and 218 (T9). Plot construction identical to that of Fig 5 of [33].

Recorded signal quality can affect decoding performance, however this relationship was not specifically evaluated in this study. To better understand the signal quality of each participant’s neural data, plots of thresholded spiking activity for each participant were taken from the start of a research day. These appear in Fig 2. Participant T5 had the largest single units across his arrays while Participant T6’s array had the least number of distinguishable single units. Further detail on the relationship between signal quality and decoding performance can be found in prior reports [29, 32, 33, 35].

### Task design

Once the decoder was calibrated, the tablet was paired with the BCI system. The technician ensured that the tablet displayed the home screen at the start of each session. Aside from ensuring that the cursor was active and under iBCI control by the participant, the technician did not otherwise intervene during tablet use. Participants used seven common applications on the tablet: an email client, a chat program, a web browser, a weather program, a news aggregator, a video sharing program, and a streaming music program. The applications used by the participants were either preinstalled with the tablet or downloaded by one of the research members from the Play Store (Google, Mountain View, CA) prior to the first day of the study. Participants were asked to launch each target application from the home screen, use as requested, and exit the program by returning to the home screen. Details of the specific tasks and programs appear in Table 1. Each participant completed the entire task design on each of three days. Tasks included periods of participant-determined actions (e.g., personal choice of typing topics) such that the number of clicks required for task completion varied across participants. For typing performance (assessed on email and chat tasks), duration was counted from the time the keyboard was activated by the participant to the time the last character or word was entered. Selections include all printed and non-printed characters (e.g., shift and delete keys). Effective characters are all printed characters that appeared as transmitted text. In addition to these structured tasks, each participant was asked in advance which additional consumer applications they would enjoy using. These applications were then downloaded from the Play Store. After completing the standard tasks, each participant proceeded to use their selected applications. On a separate day (implant day 1211 for T6 and implant day 416 for T9), T6 and T9 used the chat program to send messages to each other in real time. The research session ended at the participants’ discretion.

**Table 1:** Task and program details.

Task	Program	Source	Objective
Email	Gmail	Built-in	Check email and reply to one new message. If no new mail exists, compose an email to research staff.
Chat	Hangouts	Built-in	Have a conversation with a member of the research staff.
Web Browser	Chrome	Built-in	Perform a Google search on a topic of interest and browse through results.
Weather	Weather Underground	Play Store	Check the hourly and daily local weather forecast.
News Aggregator	News Republic	Play Store	Browse through news stories and photos of the day.
Video Sharing	YouTube	Built-in	Search for and play videos of interest.
Music Streaming	Pandora	Play Store	Play music from various radio stations.

## Results

Participants performed all tasks on each of the three research days. As an example, the task timeline for day 124 of T5 appears in Fig 1b. No technical issues surrounding decoder calibration, Bluetooth device pairing, or application crashes were encountered in any sessions.

**Fig 3:**
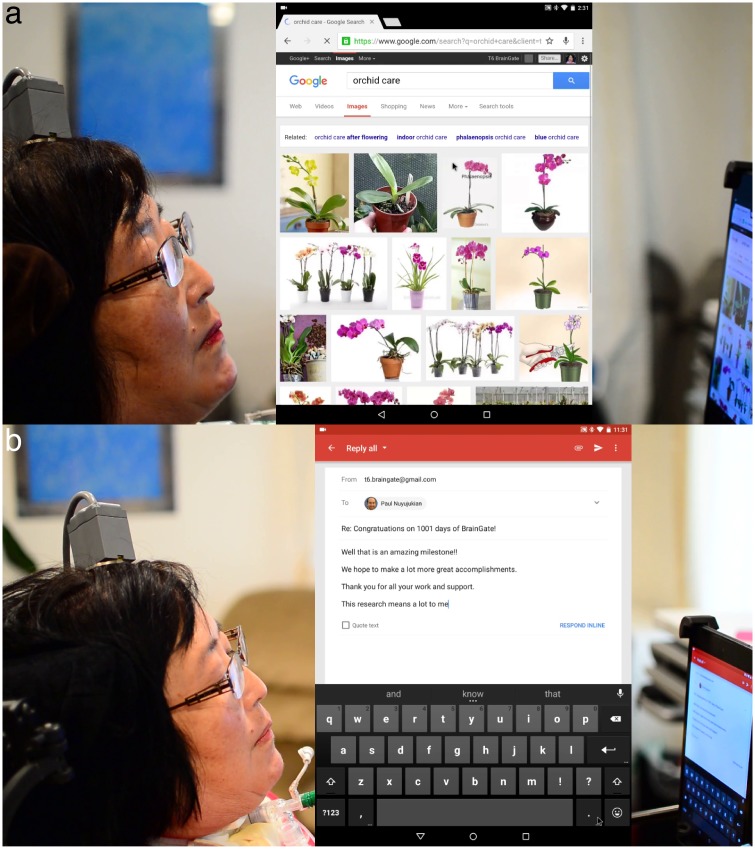
**a** T6 browsing the web. Overlay in center of the image is a screen capture of the tablet. **b** T6 composing an email (trial day 1001). Both images are taken from S1 Video.

**Fig 4:**
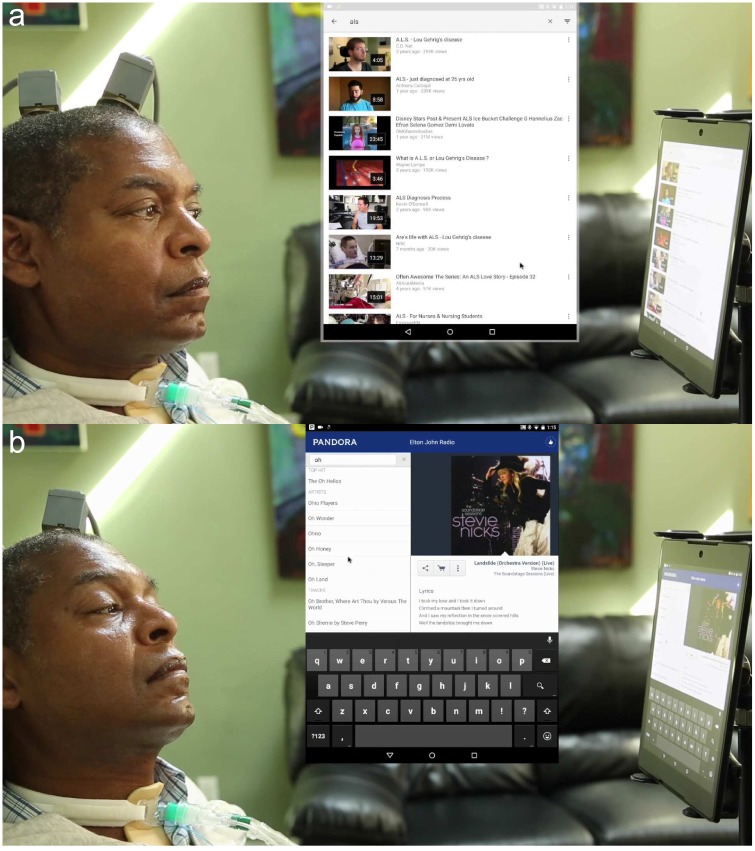
**a** T9 performing a video search. **b** T9 searching for artists from a music streaming program. Both images are taken from S2 Video.

**Fig 5:**
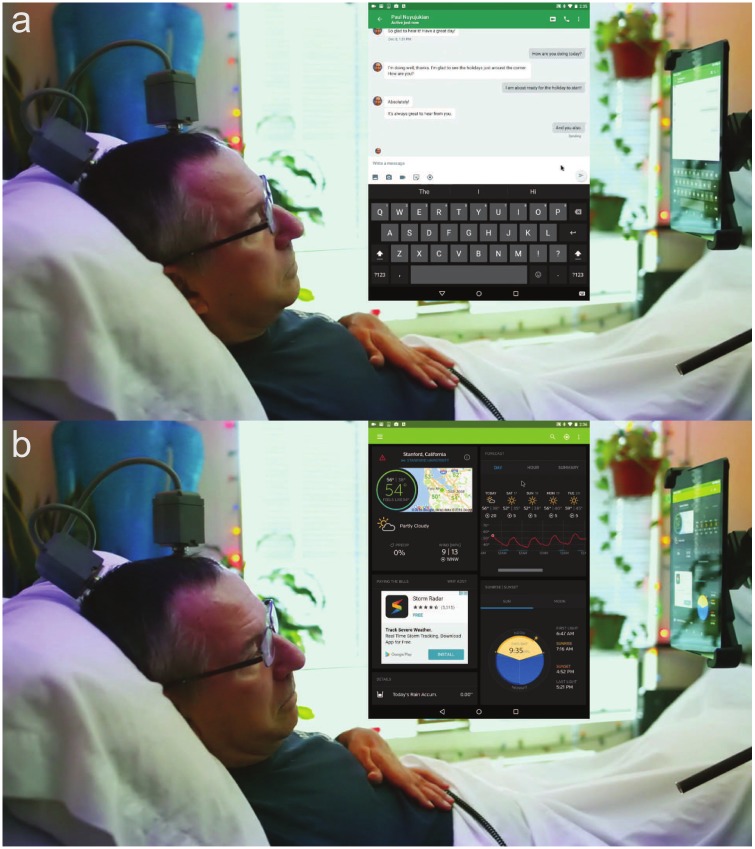
**a** T5 chatting in real time with research staff. **b** T5 checking the forecast. Both images are taken from S3 Video.

The mean time required to complete all seven tasks was 15.4 minutes for T6 (Fig 3 and S1 Video), 33.5 minutes for T9 (Fig 4 and S2 Video), and 19.8 minutes for T5 (Fig 5 and S3 Video—see Table 2 for details). When the task was interactive (e.g., email or chat), participants communicated with members of the research staff. On independent open-ended tasks (e.g., web and video searches), topics were chosen by the participants, drawing from their own interests. Variation in the number of clicks per minute across tasks reflected variation in both choice of text entry and choice of button selections.

**Table 2:** Task usage. Table of tasks in order of use, duration, and click rate of each participant on each research day. Last row for each participant includes summary statistics across all days.

T6					T9					T5				
Day	Task	Duration (MM:SS)	Total Chcks	Clicks per min	Day	Task	Duration (MM:SS)	Total Clicks	Clicks per min	Day	Task	Duration (MM:SS)	Total Clicks	Clicks per min
1013	Email	2:47	34	12.2	218	Email	7:35	60	7.9	121	Email	2:53	22	7.62
	Chat	4:15	46	10.8		Chat	9:46	90	9.2		Chat	5:33	60	10.8
	Web	1:33	17	11.0		Web	3:39	24	6.6		Weather	0:49	6	7.2
	Weather	0:26	4	9.2		Weather	1:16	6	4.7		News	1:23	6	4.3
	News	1:01	9	8.9		News	2:18	11	4.8		Web	3:13	30	9.3
	Music	0:59	6	6.1		Music	1:05	7	6.5		Music	2:29	4	1.6
	Video	1:30	14	9.3		Video	9:34	24	2.5		Video	1:40	12	7.2
1018	Email	1:31	23	15.2	222	Email	8:45	7	8.0	124	Email	2:57	34	11.5
	Chat	6:37	85	12.8		Chat	7:24	62	8.4		Chat	4:48	74	15.4
	Weather	0:28	5	10.7		Weather	0:52	6	6.9		Weather	0:28	4	8.4
	News	1:22	9	6.6		News	2:22	12	5.1		News	3:29	18	5.2
	Web	1:41	18	10.7		Web	4:47	39	8.2		Web	1:13	10	8.2
	Music	1:26	13	9.1		Music	2:44	9	3.3		Music	1:23	4	2.9
	Video	1:45	14	8.0		Video	3:15	8	2.5		Video	4:34	20	4.4
1034	Email	4:08	39	9.4	225	Email	8:14	115	14.0	140	Email	5:02	3	6.8
	Chat	7:55	92	11.6		Chat	9:42	84	8.7		Chat	6:39	98	14.7
	Weather	0:38	5	7.9		Weather	1:44	7	4.0		Music	1:25	9	6.4
	News	0:41	5	7.3		News	2:16	10	4.4		News	2:44	11	4.0
	Web	1:53	13	6.9		Web	8:29	31	3.7		Weather	0:40	5	7.5
	Music	1:14	6	4.9		Music	1:07	6	5.4		Video	3:02	11	3.6
	Video	2:28	14	5.7		Video	3:48	8	2.1		Web	2:40	28	10.5
all		46:18	471	10.2	all		100:42	689	6.8	all		59:22	548	9.2

We estimated typing rates during use of applications in which a significant portion of time was spent entering text (i.e., email and chat). Across each participant’s three research days, the mean selections per minute was 14.3, 12.0, and 22.8 for T6, T9, and T5, respectively. With the word completion/ prediction feature of the default Android OS keyboard, the mean typing rate was 24.0, 13.6, and 30.8 effective characters per minute for T6, T9, and T5, respectively (see Table 3). The word completion feature increased typing rates by 68% (T6), 13% (T9), and 35% (T5) compared to the predicted typing rate if every selection was a single character.

**Table 3:** Typing performance. Table of typing performance broken down by research day and task. Typing performance was assessed on email and chat tasks. Duration was counted from the time the keyboard was activated by the participant to the time the last character or word was entered. Selections include all printed and non-printed characters (e.g., shift and delete keys). Effective characters are all printed characters that appeared as transmitted text. Correction rate is the percent of selections that comprise the backspace button. Last row for each participant includes summary statistics across all days.

	Day	Task	Duration	Selections per min	Effective chars per min	Percent autocompleted	Correction rate (%)
T6	1013	Email	2:03	13.1	23.9	65	0.0
		Chat	2:33	15.7	32.9	70	0.0
	1018	Email	1:16	15.0	29.2	73	5.3
		Chat	4:57	17.8	26.9	45	3.4
	1034	Email	2:23	10.9	18.5	73	11.5
		Chat	6:43	12.5	19.4	35	6.0
T6	all		19:55	14.3	24.0	69	4.2
T9	218	Email	5:20	8.3	14.4	67	4.5
		Chat	7:18	10.5	13.7	62	14.3
	222	Email	5:26	10.7	12.3	34	6.9
		Chat	3:13	14.0	15.5	52	13.3
	225	Email	7:12	14.3	12.6	57	30.1
		Chat	4:58	14.9	13.9	71	31.1
T9	all		33:27	12.0	13.6	58	19.2
T5	121	Email	0:32	20.9	39.9	66	9.0
		Chat	2:08	24.3	38.8	88	0.0
	124	Email	1:07	22.3	21.4	50	12.0
		Chat	3:02	21.1	21.4	50	3.1
	140	Email	2:46	25.3	29.6	75	11.4
		Chat	2:54	21.7	38.0	71	22.2
T5	all		12:29	22.8	30.8	70	9.8

**Fig 6:**
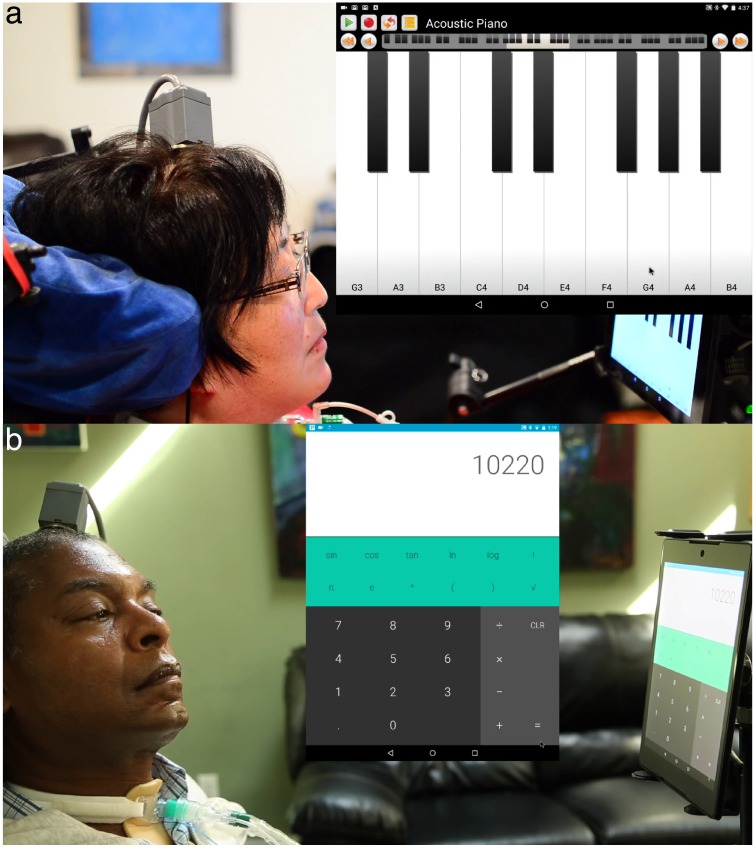
Tasks of interest. **a** T6 playing music with a keyboard application. **b** T9 using a calculator application. Both images are taken from S4 Video.

We also asked each participant what additional programs they would like to use on the tablet. Among the programs and games T6 explored, she asked to play music on a keyboard application (Fig 6a and S4 Video). On another day, T6 used the interface to shop online and purchase groceries (Amazon application, downloaded from Play Store). T9 used a calculator program (Fig 6b and S4 Video) and a word processing program. In these unstructured writing sessions, he averaged 16.7 correct characters per minute with 64% of his text autocompleted. T5 used the tablet to send text messages to friends, family, and the research staff through the Google Voice program.

Additionally, T6 and T9 used the tablet to write messages to each other in real time through an instant messaging program (S5 Video). This session corresponded to post-implant day 1211 for T6 and post-implant day 416 for T9.

Participants were asked to report on their experience using the tablet under neural control. T6 noted that “the tablet became second nature to me, very intuitive. It felt more natural than the times I remember using a mouse.” T9 said that the interface was “amazing! I have more control over this than what I normally use.” Regarding sending text messages, T5 said that he “loved sending the message. Especially because I[he] could interject some humor.”

## Discussion

Three participants, two of whom were diagnosed with ALS and were dependent on mechanical ventilators (T6, T9) and a third with tetraplegia due to spinal cord injury (T5), controlled a commercial tablet via an intuitive “point-and-click” brain-computer interface enabled by recent advances in neural decoders [24, 32, 33]. All three participants successfully used seven common computer applications on three separate days each. Additionally, each participant used other applications of their choice.

Participants navigated the user interface comfortably despite not having access to all of the gestures commonly used on a tablet (e.g., click and drag, multitouch). This precluded certain functions such as scrolling up and down on the tablet web browser. Some of these limitations would have been overcome by enabling accessibility features found in the Android OS or third-party programs. Additionally, modifying the Android OS keyboard layout as we have done in prior reports [29, 32, 33] would have likely increased typing rates. Though such changes may have enabled greater functionality and higher performance, our goal in this study was to measure the applicability of iBCI-based control on an unmodified tablet with the stock user interface, through which one can access a vast library of off-the-shelf software. Thus, our participants faced the same challenges with small selection regions (e.g., hyperlinks) that able-bodied users face. The fact that all participants could nevertheless successfully navigate the user interface demonstrates the high level of control enabled by the iBCI. Expanding the control repertoire with additional decoded signals, leveraging more optimized keyboard layouts, exploring accessibility features, and controlling other devices and operating systems are subjects of future study. Further work is needed to extend the output of the iBCI to support additional dimensions that may be used to command these advanced cursor features. For example, a click decoder with multiple transition states beyond just instantaneous click could implement click-and-hold and gestures. True multitouch input would require additional independent analog dimensions to be decoded, two per additional touchpoint.

Participants T6 and T5 also participated in our prior report on copy typing rates with iBCIs on trial days 570-621 and 56-70, respectively [33]. Comparing the best typing rates of that study with results here, we saw a decrease of 24% (T6) and 21% (T5). This is likely due to several factors, including the free-typing performed here versus the copy typing task in the prior study, and the use of a stock QWERTY keyboard layout here versus an optimized keyboard layout (OPTI-II) in the prior study. Notably, when we compare typing rates on identical keyboard layouts (QWERTY) between the studies, T6’s performance was very similar (23.9 vs 24.0 ccpm in the previous study vs. present study, respectively, with word completion/prediction) and T5’s performance decreased by 15% (36.1 vs. 30.8 ccpm) in the present study.

The findings here also demonstrate that iBCIs can be used as tools beyond simple communication. T9 used the tablet to search for information and videos on ALS. The interface also facilitated entertainment and pursuit of hobbies. He would often leave the streaming music program running in the background while using the iBCI to use other applications. T6 frequently used the web browser application to search for information about her hobbies. T5 enjoyed messaging friends and family and watching videos, sending his first text messages ever via the iBCI in this study. Additionally, the iBCI was used as a tool for self-expression, both through writing and music. As a musician, T6 enjoyed using the musical keyboard. In fact, this was one of her earliest requests of the research team when she joined the study: to play music again. Providing her with a music keyboard interface on the tablet computer was as simple as installing an application from the Internet. One strength of the approach in this study is leveraging a mature, industry-scale suite of software. Particularly for AAC systems, custom user interfaces often limit the scope of applications available to the user. By seamlessly integrating the iBCI with a mature computing platform, participants used many programs and features (e.g., built-in, advanced language modeling for improved text entry performance) that would have otherwise been impractical to implement by the research team.

## Conclusion

To our knowledge, this is the first use of a commercial, unmodified general-purpose computing device and associated programs through a BCI by people with paralysis. The performance achieved here is high enough to be useful for individuals unable to control computing devices using conventional, manual input devices. We also note that these studies were conducted 2.75 years (T6), nine months (T9), and four months (T5) after implantation of the electrode arrays. This provides additional evidence that iBCIs can potentially provide high-quality control for extended periods of time [26, 27]. It is also notable that intracortical neural signals derived from the precentral gyrus (motor cortex) allowed for effective iBCI control, providing additional evidence of volitionally modulated neuronal activity in this region in at least some people with advanced ALS [31–33]. Ongoing research is focused on creating systems that provide not only demonstrations of feasibility, but the potential for robust, independent BCI-enabled use of ubiquitous communication technologies. This study is another step towards the increasing utility of iBCIs as potential assistive, communication, education, environmental control, and entertainment devices for individuals with paralysis. With continued iBCI research and development, these data also suggest that maintenance of communication may be possible, using appropriate technologies, through the progression of ALS and perhaps even through what would otherwise become a locked-in state [37].

## Supporting information

S1 Video Participant T6—Web browsing & email. (MP4)

S2 Video Participant T9—Video search & streaming music. (MP4)

S3 Video Participant T5—Chat & weather. (MP4)

S4 Video Tasks of interest—T6 piano & T9 calculator. (MP4)

S5 Video Cross-coast iBCI chat between T6 and T9. (MP4)
